# Remarks on the London Museum, Brydges Street, Covent Garden

**Published:** 1807-11

**Authors:** J. Laskey

**Affiliations:** Captain 21st Militia, Edinburgh


### *To the Editors of the Medical and Physical Journal.*

### *Remarks on the London Museum, Brydges Street, Covent Garden.*

Gentlemen,

FROM reading Mr. Parkinson's observations on the British Encrinites, and other mineralized remains of the organic world, inserted in one of the magazines of last month, I learn with infinite pleasure that the Institute of Natural History, established in the metropolis last April, still remains open to public inspection. This was originally proposed, I recollect, to be open only during the spring season annually; and as I reside in Scotland, the total silence of the literary journals and channels of scientific intellgence, respecting it, led me to think it must be closed, till accidentally taking up a magazine of last month, month, I was convinced to the contrary; and then, I must say, I felt some astonishment that it had so long escaped the notice of your interesting Miscellany.

As an admirer of the works of Nature, I should consider myself wanting in candour to withhold my tribute of approbation from such a noble undertaking, or to neglect any opportunity, should it be necessary, to direct the attention of the curious to this Museum. The endeavour to establish such a National Academy of the Natural History of the Country, is entitled to every praise we can bestow, and reflects so much credit on the liberality, judgment, assiduity, and laudable spirit of Mr. Donovan, the proprietor, that I am sure every one must agree with me in considering it one of the greatest and most successful attempts ever made in this country for the promotion of Science.

When I was in London, early in the summer, my visits to this Museum were frequently repeated, and I always saw it with additional delight and pleasure. From my attachment to Natural History in general, I have at various times had an opportunity of seeing almost every Cabinet and Museum, public as well as private, of any celebrity in this country; and I am confident in saying, that so far from any one of these being comparable with this, the whole of them, added together, would not form a collection of British Natural History, by any means so extensive, valuable, or instructive. I consider the divisions of birds and fishes the only perfect collections known. The organic remains of the ancient world consist of the most illustrative specimens; and I cannot help observing further, in every other department, objects of the greatest variety occur. Considering as I do the present collection to be enriched with the choicest British productions of natural curiosity in the animal and mineral kingdoms, obtained at the dispersion of the Leverian and other great Collections, (with which I was well acquainted) besides thousands of valuable and inestimable articles, I have never seen the like of elsewhere, have no scruple in saying it would be impossible at this time for any Collector, possessing the most unwearied attention, sanguine wish, and unlimited purse, to form another Collection equal to that now before the public, under the appellation of the London Museum.

The observations I have offered, may, and no doubt will, dispose others to enter more largely into the plan and merits merits of this Institution, and which, for the benefit of Science, ought to be made better known.

I am, &c.

*Oct.* 5, 1807. J. LASKEY,

Captain 21st Militia, Edinburgh.

